# Pixel or Paper? Validation of a Mobile Technology for Collecting Patient-Reported Outcomes in Rheumatoid Arthritis

**DOI:** 10.2196/resprot.5631

**Published:** 2016-11-16

**Authors:** Oscar Massimiliano Epis, Cinzia Casu, Laura Belloli, Emanuela Schito, Davide Filippini, Marina Muscarà, Maria Giovanna Gentile, Paula Carina Perez Cagnone, Chiara Venerelli, Massimo Sonnati, Irene Schiavetti, Eleonora Bruschi

**Affiliations:** ^1^Rheumatology UnitAzienda Socio-Sanitaria Territoriale Grande Ospedale Metropolitano NiguardaMilanoItaly; ^2^Hippocrates Sintech SrlGenovaItaly; ^3^Hippocrates Research SrlGenovaItaly

**Keywords:** validation, rheumatoid arthritis, PROs, monitoring, electronic device, tablet, questionnaire, paper

## Abstract

**Background:**

In the management of chronic disease, new models for telemonitoring of patients combined with the choice of electronic patient-reported outcomes (ePRO) are being encouraged, with a clear improvement of both patients’ and parents’ quality of life. An Italian study demonstrated that ePRO were welcome in patients with rheumatoid arthritis (RA), with excellent matching data.

**Objective:**

The aim of this study is to evaluate the level of agreement between electronic and paper-and-pencil questionnaire responses.

**Methods:**

This is an observational prospective study. Patients were randomly assigned to first complete the questionnaire by paper and pencil and then by tablet or in the opposite order. The questionnaire consisted of 3 independent self-assessment visual rating scales (Visual Analog Scale, Global Health score, Patient Global Assessment of Disease Activity) commonly used in different adult patients, including those with rheumatic diseases.

**Results:**

A total of 185 consecutive RA patients were admitted to hospital and were enrolled and completed the questionnaire both on paper and on electronic versions. For all the evaluated items, the intrarater degree of agreement between 2 approaches was found to be excellent (intraclass correlation coefficient>0.75, *P*<.001).

**Conclusions:**

An electronic questionnaire is uploaded in a dedicated Web-based tool that could implement a telemonitoring system aimed at improving the follow-up of RA patients. High intrarater reliability between paper and electronic methods of data collection encourage the use of a new digital app with consequent benefit for the overall health care system.

## Introduction

Patient-reported outcomes (PROs), defined by the US Food and Drug Administration as “any report of the status of a patient's health condition that comes directly from the patient,” are becoming more and more common in the medical field, with an increasing improvement of dedicated software solutions for electronic capturing of data [1]. Furthermore, general advantages of using online formats compared to paper ones were already highlighted in the early 90s [2] and confirmed by further studies [3,4].

At present, the long-term disease monitoring of patients at home exemplifies the most promising application of telemonitoring technology for supplying cost-effective quality care [5]. Therefore, especially in the management of chronic disease, new models for telemonitoring of patients combined with the choice of electronic PRO (ePRO) are being encouraged, allowing a self-managing of patient care during all treatment phases, with a clear improvement not only of patients’ but also of parents’ quality of life, as reported in a recent pediatric study [6].

In the field of rheumatoid arthritis (RA), the validity and effectiveness of PRO data in addition to the standard clinical practice for the intensive care of the patients is well documented [7,8]. A recent systematic review from Johns Hopkins University assesses the frequency and the analyzed domains of PRO used in recent RA studies by collecting and summarizing data from 250 articles [9]. The first Italian study demonstrated that ePRO were welcome in patients with RA, with high levels of agreement between paper and electronic data and good reliability findings [10].

In 2010, the rheumatology unit of Azienda Socio-Sanitaria Territoriale Grande Ospedale Metropolitano Niguarda introduced a computer touch screen–based technology with the aim to collect and manage clinical data during the examinations of RA patients. In recent years, the daily medical practice has implemented this system and the assessment of PROs [11].

According to the outcome research guidelines proposed by the International Society for Pharmacoeconomics and Outcomes Research, ePRO questionnaires should provide comparable or better data than a paper questionnaire, and measurement of the difference between the 2 data-gathering approaches is a necessary validation method [12].

The task of this study is to compare electronic and paper-and-pencil questionnaire responses and verify that the ePRO supported by the use of innovative mobile technologies can be widely used in a program of tailored telemonitoring of RA patients.

## Methods

### Study Design and Sample Size Calculation

**Figure 1:**
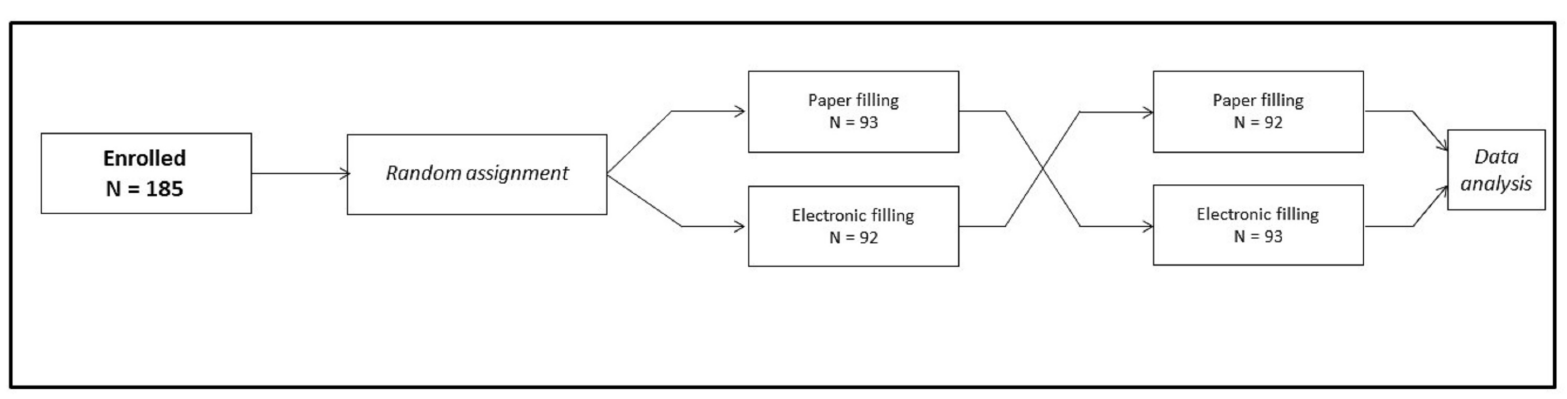
Study design.

This is an observational prospective study. All the patients in the study were randomly assigned to first complete the questionnaire by paper and pencil and then by tablet or in the opposite order (Figure 1). After both questionnaires were completed, physicians asked the patients to indicate their preferred version and comment on the accuracy of the paper in terms of it being easy to read and interpret. These additional data were collected together with demographics in a dedicated database.

Sample size was calculated basing on the literature regarding the estimates for sample size requirements for reliability studies using an intraclass correlation coefficient (ICC) [13]. In particular, assuming a possible 25% dropout rate [14], 185 patients were sufficient to detect an expected reliability of 0.8 against an acceptable reliability of 0.7, with an 85% power and a significance level of .05.

### Ethics, Consent, and Permissions

There was no need for ethical approval for this study. All the patients signed an informed consent form.

### Recruitment

All patients aged 18 to 90 years who attended the Azienda Socio-Sanitaria Territoriale Grande Ospedale Metropolitano Niguarda between July and September 2013 and met the American College of Rheumatology criteria for RA [15] were considered for enrollment.

### Questionnaire

The questionnaire consisted of 3 independent self-assessment visual rating scales, commonly used in different adult patients including those with rheumatic diseases [16].

1. Visual Analog Scale (VAS) pain [17]: RA patients reported the degree of their pain in a scale ranging from 0 (no pain) to 100 mm (worst imaginable pain).

2. Global Health (GH) score: patients reported the level of impact of the RA disease on their global health with a value ranging from 0 (no effect) to 100 mm (maximum effect).

3. Patient Global Assessment of Disease Activity (PGA) [18]: patients answered the question “In the last week, how active would you define your rheumatic condition?” on a 0 to 100 mm scale ranging from “not active at all” to “extremely active” as anchors. 

Patients received both a device with an app version and a paper version. In the first case, they had to touch the visual scale on the screen of the electronic device at the same point corresponding with the status of their response; once the selected questionnaire was completed and saved, the relative score was automatically calculated. For the paper version, patients were asked to place a perpendicular line in each scale at the point which best matched with the status of their response. The score was determined by measuring with a ruler the distance (mm) between the “0” dash and the patient's mark.

### App Details

Electronic questionnaires are part of a dedicated Web-based tool that helps physicians managing patients affected by RA. Every piece of information is sent to a cloud system, and the security of the communication is ensured by authentication routines.

Therefore, the platform is accessible both through a link on the Web and through a dedicated app for the tablet. The Web form, accessible through a link, represents the core of the app and includes “entry,” “search,” and “edit” data functions. All data input from the tablet or Web are stored in a dedicated database in the main repository of the system.

At first, the physicians can create detailed and accurate patient profiles by filling in demographic and clinical assessments. Once the profile is created, patients receive a username and password to access the self-examination module and fill in the PROs.

The tool allows real-time data gathering, with data displayed in various ways including pictograms, balloon chart, and chromatic scales. All information is accessible both through the Web site and tablet app, which allow full access to the patients’ data as well as direct access to the report printing functionalities.

### Statistical Analysis

The normality of continuous variables was checked by the examination of histograms and confirmed by the Kolmogorov-Smirnov test. All nonnormally distributed data were ranked before further analysis. Demographic characteristics were summarized as count and percentage, mean and standard deviation, and median with range. Any difference between the orders of the questionnaire administration was assessed by using a chi-square test or Fisher's exact test for categorical data and independent sample *t* test for continuous data.

To evaluate possible order and format effects, any difference between paper and electronic results of each evaluated item was analyzed by an analysis of variance test for repeated measures with format as repeats and order of administration as factor.

For all the items, the level of agreement between the responses of electronic and paper formats was estimated with the ICC, expressed with 95% confidence interval. Fleiss recommendations [19] were followed to identify ICC cut-offs.

A Bland-Altman plot [20] was used to graphically confirm the results and visualize the concordance degree. This chart, for each subject, plots the difference between the 2 measurements (the *y* axis) as a function of the mean of the same values (the *x* axis). All statistical tests were 2-sided and the significance level (alpha error) was set at .05.

## Results

A total of 185 consecutively admitted patients to hospital (100% recruitment rate), aged between 26 and 83 years, with a diagnosis of RA were invited to participate in the study.

All enrolled patients completed the questionnaire both on paper and on electronic versions. Randomization process was conducted without any statistically significant difference in the baseline characteristics between 2 groups (Table 1).

Likewise, no difference concerning the information on the quality of the questionnaire was revealed (Table 2).

For all the items, no significant main effects of order, format, or the interaction effect of both was observed, indicating that the order of completion did not matter (Table 3).

For all the evaluated items, the intrarater degree of agreement between paper and electronic responses was found to be excellent (ICC>0.75, *P*<.001) (Table 4).

**Figure 2:**
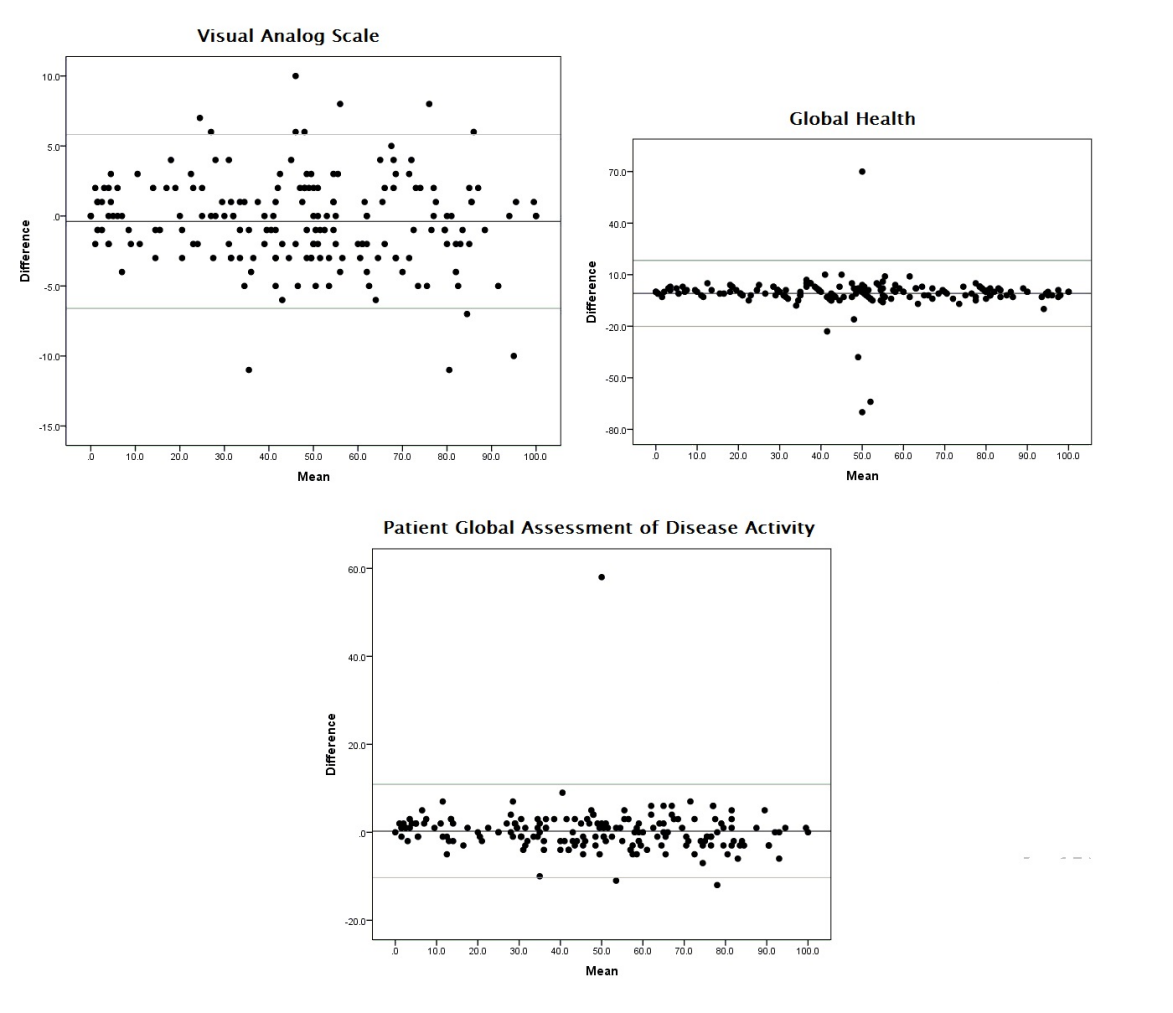
Bland Altman plots.

The same results were graphically confirmed by the Bland-Altman plots (Figure 2).

**Table 1:** Baseline characteristics of the sample.

Demographic data		Total N=185	First paper then electronic version n=93	First electronic then paper version n=92	*P* value
Age (years), mean (SD)		59.5 (12.1)	59.5 (11.0)	59.4 (13.1)	.96
**Sex, n (%)**					
	Female	155 (83.8)	82 (88.2)	73 (79.3)	
	Male	30 (16.2)	11 (11.8)	19 (20.7)	.15
**Nationality, n (%)**					
	Italian	166 (89.7)	83 (89.2)	83 (90.2)	
	Foreign	19 (10.3)	10 (10.8)	9 (9.8)	>.99
**Employment, n (%)**					
	Retired	52 (28.1)	26 (28.0)	26 (28.3)	
	Workman	25 (13.5)	13 (14.0)	12 (13.0)	
	Employee	55 (29.7)	28 (30.1)	27 (29.3)	
	Housewife	53 (28.6)	26 (28.0)	27 (29.3)	.99
**Education level, n (%)**					
	Lower school	32 (17.3)	16 (17.2)	16 (17.4)	
	Middle school	60 (32.4)	30 (32.3)	30 (32.6)	
	High school	75 (40.5)	39 (41.9)	36 (39.1)	
	Degree	18 (9.7)	8 (8.6)	10 (10.9)	.95

**Table 2:** Data regarding the quality of the questionnaires.

Quality of the questionnaires		Total N=185 n (%)	First paper then electronic version n=93 n (%)	First electronic then paper version n=92 n (%)	*P* value
**Accuracy of the paper version**						
	No	34 (18.4)	13 (14.0)	21 (22.8)	
	Yes	151 (81.6)	80 (86.0)	71 (77.2)	.17
**Preferred version**						
	Electronic	176 (95.1)	88 (94.6)	88 (95.7)	
	Paper	9 (4.9)	5 (5.4)	4 (4.3)	>.99

**Table 3:** Summary of *F* values from 2-way analyses of variance for format and order effect (N=185).

ANOVA	*F* values	*P* value
Visual Analog Scale	0.93	.34
Global Health Scale	0.08	.78
Patient Global Assessment	1.59	.21

**Table 4:** Agreement degree in the response questionnaires between 2 formats (N=185).

Visual Rating Scales	Paper version Mean (SD) Median (min-max)	Electronic version Mean (SD) Median (min-max)	ICC (95% CI)	*P* value
Visual Analog Scale	46.7 (25.9) 49.0 (0.0-100.0)	47.1 (26.3) 48.0 (0.0-100.0)	0.996 (0.995-0.997)	<.001
Global Health	48.8 (26.3) 50.0 (0.0-100.0)	49.7 (26.7) 50.0 (0.0-100.0)	0.959 (0.945-0.969)	<.001
Patient Global Assessment	47.8 (25.7) 50.0 (0.0-100.0)	47.5 (26.1) 49.0 (0.0-100.0)	0.988 (0.984-0.991)	<.001

## Discussion

### Principal Findings

In the modern clinical practice, fast and reliable collection of clinical data is an important need. The widespread use of mobile technologies throughout the world has also involved the medical field, where the use of information technology products and services allows access to health care, both containing costs and improving the quality of data and clinical outcomes.

As reported by the World Health Organization, mHealth or mobile health is a “medical and public health practice supported by mobile devices, such as mobile phones, patient monitoring devices, personal digital assistants, and other wireless devices” [21].

This study proved that a new approach for telemonitoring, where desktop applications are fully integrated with external mobile devices, could play an important role in the patient follow-up. Response rate was 100% for both methods, Internet-based electronic questionnaires comply with the traditional paper formats, and the app is considered easy to use (electronic version is defined as the preferred choice by most patients).

In particular, for all the investigated items, no significant differences (by considering also any order effects) between the 2 approaches were found, and these findings are in agreement with previous studies reporting none or very few differences between computerized and paper-and-pencil assessments [22,23].

Furthermore, it is important to stress that ePRO can provide a valuable source of information. Data can be collected at any time of the day according to the wishes of the patient. In this way, the database can be fed continuously over time, providing the physicians useful tools for better defining strategies to deal with a long-lasting (chronic) disease. These findings have shed some light on the significant value to the patient, but they mostly highlight the benefit that the overall health care system can enjoy from this new digital app.

### Limitations

These first results are applicable to RA patients, but further studies are recommended because the comparison between 2 approaches in other populations and settings has not yet been studied.

Furthermore, performed comparisons should be weighted by considering the low amount of collected data, in this case 3 independent single items. It would be interesting to gather data in order to study more complex measures of outcomes.

Sufficient privacy and security should be guaranteed: data transferred to a health care provider may be subject to hacking. A disaster recovery system should be ready at all times, and technical support for corrupted or erased data must be set up.

Finally, it is important to point out that rheumatology specialist care should never be substituted: telemonitoring systems are to be considered as an important challenge in the modern times, but they are complementary tools and not alternatives in the routine medical practice. 

## Multimedia Appendix 1

Visual Analog, Global Health, and Patient Global Assessment scales.

## Abbreviations

ePRO: electronic patient-reported outcome
GH: Global Health
ICC: intraclass correlation coefficient
PGA: Patient Global Assessment of Disease Activity
PRO: patient-reported outcome
RA: rheumatoid arthritis
VAS: Visual Analog Scale
