# Supplementary figures and images for: Pixel or Paper? Validation of a Mobile Technology for Collecting Patient-Reported Outcomes in Rheumatoid Arthritis
Source: JMIR Res Protoc. 2016 Nov 16;5(4):e219. doi: 10.2196/resprot.5631 (PMC5131193; doi:10.2196/resprot.5631)

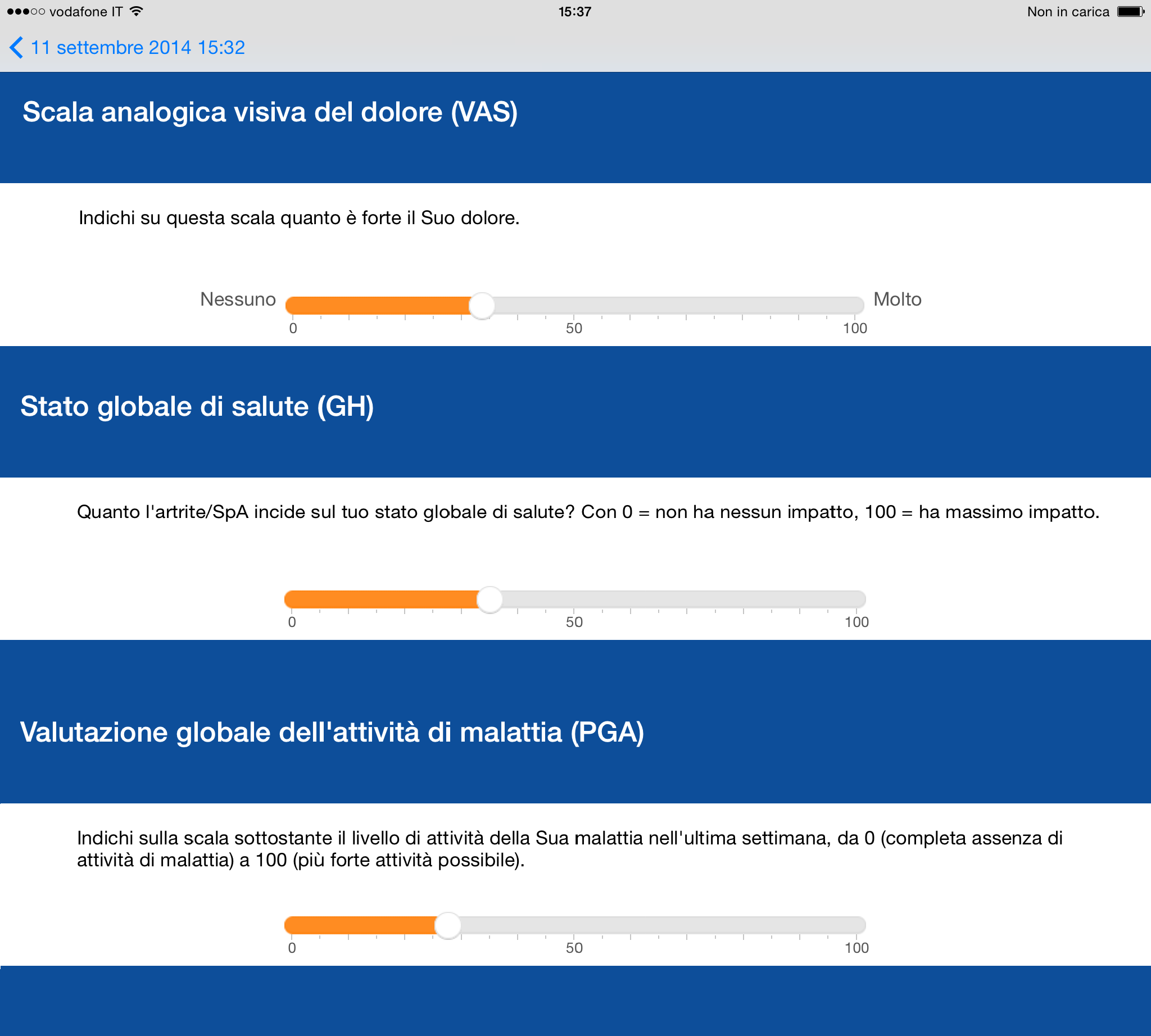

Supplement: Supplementary file 1 [file resprot_v5i4e219_app1.png]
